# Editorial: Clinical management of *Helicobacter pylori* infections

**DOI:** 10.3389/fmed.2024.1458830

**Published:** 2024-07-18

**Authors:** Raffaele Pellegrino, Amin Talebi Bezmin Abadi, Antonietta Gerarda Gravina

**Affiliations:** ^1^Department of Precision Medicine, University of Campania Luigi Vanvitelli, Naples, Italy; ^2^Department of Bacteriology, Tarbiat Modares University, Tehran, Iran

**Keywords:** *Helicobacter pylori*, gastric xanthelasma, antibiotic resistance, probiotics, *Saccharomyces boulardii*

## What new insights into *Helicobacter* (*H*.) *pylori* epidemiology have emerged from this topic?

It is known that *H. pylori* can be associated with various gastric and extra-gastric disorders in addition to typical manifestations such as gastric atrophy and *H. pylori*-related tumors (such as gastric MALToma) (1, 2). One of the studies included in current Research Topic focused on one of the tumor-like lesions that can be associated and are partly pinpointed to be the result of chronic gastritis caused by *H. pylori*, as well as other conditions (i.e., diabetes or hyperlipidaemia), namely gastric xanthelasma (3).

Feng et al. conducted a large-scale retrospective study weighing the risk correlations between gastric/esophageal/duodenal xanthelasma and various gastroesophageal conditions, including superficial gastritis, *H. pylori* infection, gastric polyps, and esophageal and gastric neoplastic diseases. Among nearly 70,000 subjects, 1.77% presented with gastric xanthelasma, confirming its rarity. This population exhibited a higher prevalence of atrophic gastritis, intestinal metaplasia, gastric cancer, and *H. pylori* infection, identified as predictors in multivariate logistic regression. In contrast, esophageal and duodenal xanthelasma did not show significant distributional differences concerning these gastroesophageal pathologies.

The success in various therapeutic regimens of *H. pylori* infection still relies on antibiotic combinations (2, 4), making this condition highly impactful by the rising antibiotic resistance observed worldwide (5). Therefore, it is crucial to profile trends in antibiotic resistance related to *H. pylori* nationally to propose effective antimicrobial regimens and mitigate the emergence of further antibiotic-resistant profiles.

Zhou et al. conducted a multicentre retrospective study involving approximately 1,600 pediatric patients who underwent endoscopy based on gastrointestinal symptoms, resulting in around 500 patients testing positive for *H. pylori* infection. The authors assessed resistance rates to clarithromycin (39.8%), metronidazole (78.1%), and levofloxacin (20.2%), as well as dual resistance rates (primarily for clarithromycin and metronidazole, nearly 20%). The authors achieved an eradication rate exceeding 90% using tailored treatments guided by these resistance rates. Conclusively, further prospective studies are necessary to validate and consolidate this approach.

## What insights into the diagnosis of this infection? The non-invasive *H. pylori* diagnostic tests maintain good performance even in the elderly

Antibiotic combinations and acid-suppressive therapy for *H. pylori* eradication may raise concerns regarding the safety of such antimicrobial regimens in vulnerable populations, such as those with significant comorbidities and the elderly. This stems from a specific risk of adverse events associated with such treatments, with heightened concern, for example, in the elderly population, although some regimens, including triple therapy, have shown reassuring profiles (6). On the other hand, it is also essential to consider whether the diagnostic approaches used for detecting this infection in the elderly population are as practical as those in younger populations to minimize the risk of false positives or false negatives and initiate treatment only when necessary.

Omar et al. conducted a systematic review and updated meta-analysis on the diagnostic performance of non-invasive tests for *H. pylori* in the elderly population, including eight studies with approximately one thousand patients. It emerged that the *H. pylori* stool antigen test (HpSA) had a pooled sensitivity and specificity of 72.5% and 94.7%, respectively, in the elderly. The respective values for the urea breath test were 96.4% and 88.3%. These two tests thus maintain optimal diagnostic accuracy even in patients over 60 years old.

On the diametrically opposite side, still within the domain of *H. pylori* infection diagnosis, Nishino et al. provided an intriguing case report where a 15-year-old patient encountered a rare repeated urea breath test results falsely positive due to the presence of oral bacterial flora in the stomach with urease activity, not related to *H. pylori*, which likely caused this false positivity in the test.

## What are the insights for the treatment of this infection? Profiling the genetics of *H. pylori* and integrating probiotics

As previously mentioned, the increasing rate of antibiotic resistance in *H. pylori* is alarming globally (7). Therefore, a particularly relevant and clinically meaningful research area in this setting is the non-invasive profiling of resistance profiles in individual patients to tailor the most effective and suitable treatment upfront. Another research field that has emerged based on these assumptions is the investigation of primary or secondary prophylaxis of this infection through vaccines (8). However, their use is still significantly constrained by the need for evidence justifying their widespread deployment. Some strains of *H. pylori* harbor mutations that may suggest resistance to certain antibiotics. For instance, point mutations in the genes A2142G, A2143G, T2182C, and C2195T have shown high prevalence in clarithromycin-resistant strains (9, 10).

Cho et al. enrolled nearly 500 *H. pylori*-infected patients without point mutations A2142G and A2143G (detected via dual priming oligonucleotide PCR) who underwent triple therapy with clarithromycin. Eradication rates did not differ between patients receiving treatment for seven days or more, suggesting no benefit in extending treatment beyond seven days in this subgroup.

Over time, we have witnessed a massive introduction of probiotic therapy in *H. pylori* eradication regimens aimed at mitigating adverse events associated with antibiotics and acid-suppressive therapy, the primary antimicrobial regimens used (11, 12). These innovations are not limited to bacteria alone but also include the emergence of the role of fungal probiotics (13).

Chen et al. conducted a meta-analysis with a systematic review evaluating the impact of supplementing *Saccharomyces boulardii* probiotic to quadruple therapy with bismuth for *H. pylori* infection, including six randomized controlled trials with over a thousand patients. Such supplementation improved the eradication rate (87.0%) compared to quadruple therapy alone (83.3%), with a significant relative risk of 1.05 between the two comparison arms. This increase in effectiveness also translated into a lower and significant relative risk of adverse events (0.56). This study confirms that probiotic supplementation plays a role in *H. pylori* eradication, as suggested by current international guidelines (2, 4).

## Author contributions

RP: Conceptualization, Methodology, Project administration, Supervision, Validation, Visualization, Writing – original draft, Writing – review & editing. AT: Conceptualization, Methodology, Project administration, Supervision, Validation, Visualization, Writing – original draft, Writing – review & editing. AG: Conceptualization, Methodology, Project administration, Supervision, Validation, Visualization, Writing – original draft, Writing – review & editing.
